# The role of PI3K signaling pathway in Alzheimer’s disease

**DOI:** 10.3389/fnagi.2024.1459025

**Published:** 2024-09-27

**Authors:** Jingying Pan, Qi Yao, Yankai Wang, Suyan Chang, Chenlong Li, Yongjiang Wu, Jianhong Shen, Riyun Yang

**Affiliations:** ^1^Department of Histology and Embryology, Medical School of Nantong University, Nantong, China; ^2^Department of Neurosurgery, Affiliated Hospital of Nantong University, Nantong, China

**Keywords:** Alzheimer’s disease, PI3K, tau protein, amyloid-*β*, Akt, GSK3

## Abstract

Alzheimer’s disease (AD) is a debilitating progressively neurodegenerative disease. The best-characterized hallmark of AD, which is marked by behavioral alterations and cognitive deficits, is the aggregation of deposition of amyloid-beta (Aβ) and hyper-phosphorylated microtubule-associated protein Tau. Despite decades of experimental progress, the control rate of AD remains poor, and more precise deciphering is needed for potential therapeutic targets and signaling pathways involved. In recent years, phosphoinositide 3-kinase (PI3K) and Akt have been recognized for their role in the neuroprotective effect of various agents, and glycogen synthase kinase 3 (GSK3), a downstream enzyme, is also crucial in the tau phosphorylation and Aβ deposition. An overview of the function of PI3K/Akt pathway in the pathophysiology of AD is provided in this review, along with a discussion of recent developments in the pharmaceuticals and herbal remedies that target the PI3K/Akt signaling pathway. In conclusion, despite the challenges and hurdles, cumulative findings of novel targets and agents in the PI3K/Akt signaling axis are expected to hold promise for advancing AD prevention and treatment.

## 1 Introduction

**Figure 1:**
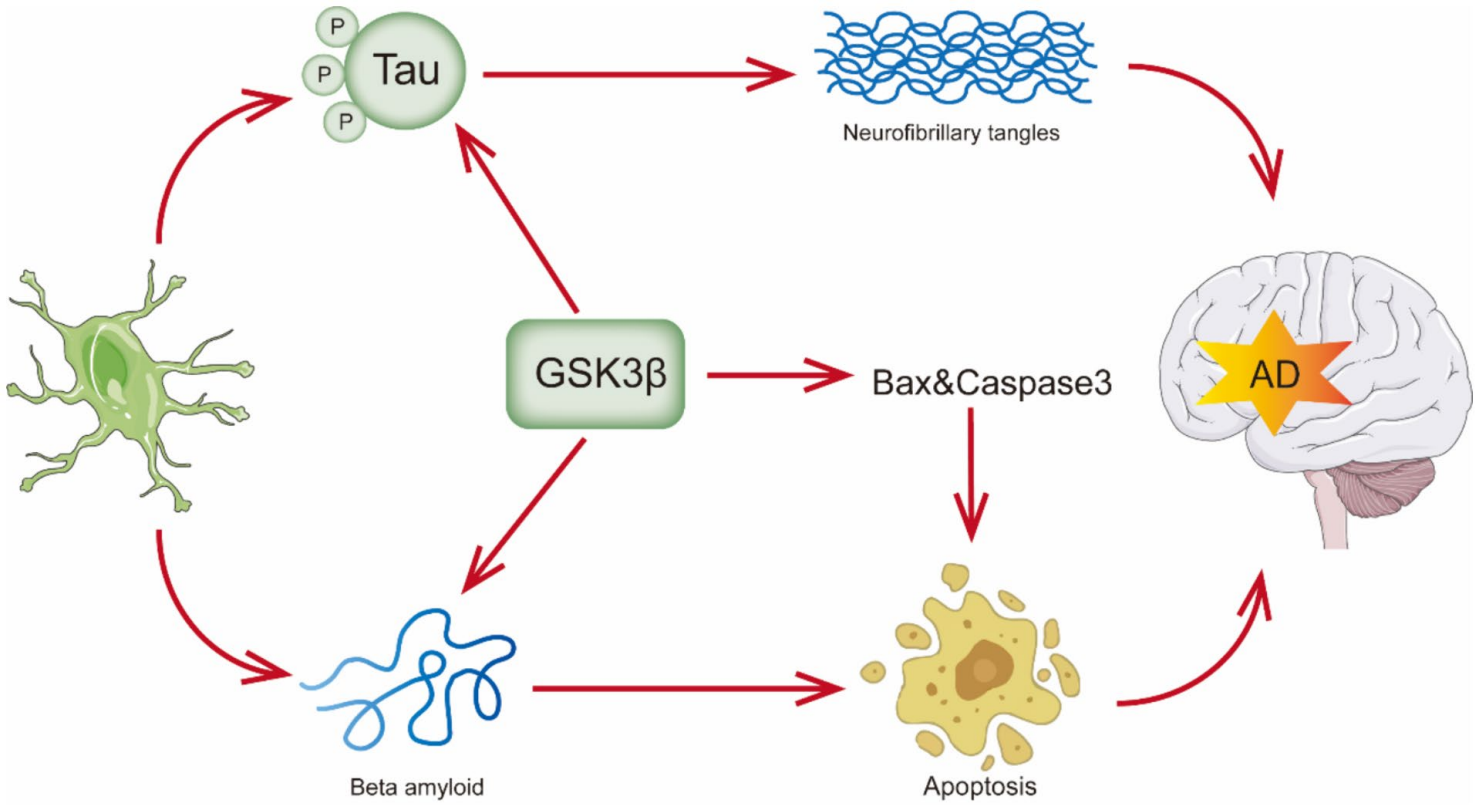
Overview of pathologic features of Alzheimer’s disease.

Progressive cognitive deficiencies, functional impairment, and behavioral abnormalities characterize AD. As it is well recognized, there are two pathological hallmarks of AD. The first is the buildup of senile plaques, which are composed of fibrils of amyloid-*β* peptide (Aβ), such as Aβ_40_ and A*β*_42_ in the central nervous system. Aβ plaque formation initiates with the aberrant cleavage of the amyloid precursor protein (APP) by β-secretase and *γ*-secretase, leading to the extracellular accumulation of Aβ, particularly the Aβ_42_ form. These peptides tend to aggregate and form plaques (Pichet Binette et al., 2024). The other pathological hallmark is the occurrence of intracellular neurofibrillary tangles (NFTs), produced by hyperphosphorylation of Tau protein’s twisted filaments (also known as *τ* protein) (Kumar and Bansal, 2022; Shefa et al., 2019). (Figure 1) PHFs, or paired helical filaments, are composed of hyperphosphorylated tau protein, which is central to the formation of neurofibrillary tangles in AD (Meng et al., 2022). The tau protein found in PHF is always hyperphosphorylated and truncated, which contains all six tau isoforms, and recent studies have shed further light on the role of tau protein hyperphosphorylation in AD pathology, emphasizing its connection to cognitive deterioration and neurodegeneration (Li et al., 2020; Basheer et al., 2023). In addition to harming the quality of life for the elderly, AD causes a great deal of financial and psychological hardship for families, society, and even the global economy, with a projected $345 billion in costs in 2023 (Alzheimer’s and Dementia, 2023). By 2050, AD is projected to affect 152 million people worldwide (Cummings et al., 2019). Therefore, in the 21st century, AD is swiftly rising to the top of the list of diseases in terms of expense, mortality, and burden.

**Figure 2:**
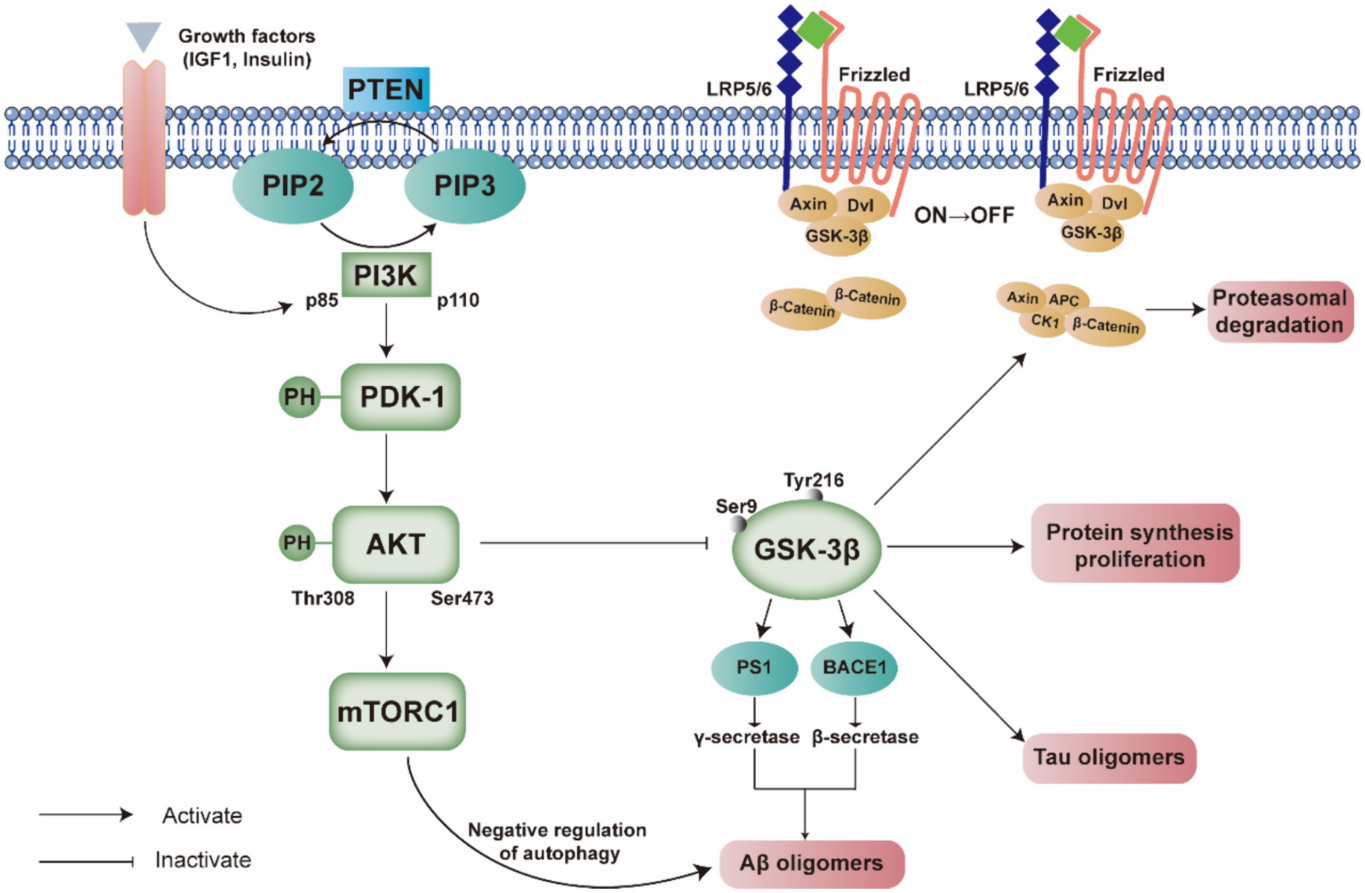
Comprehensive illustration of the interplay between the PI3K/PDK/Akt signaling pathway and the pathogenesis of Alzheimer’s disease. This figure delineates the activation and regulation of key components within the pathway, such as PI3K p85 and p110 subunits, PDK-1, and AKT, and their downstream effects on molecules such as GSK-3β, mTORC1, and the Wnt signaling components. The legend emphasizes the reciprocal modulation between these signaling molecules and the formation of Aβ and Tau oligomers, which are hallmarks of Alzheimer’s disease. The pathway’s dysregulation is implicated in the disease’s progression, affecting protein synthesis, proliferation, and autophagy. The figure also highlights the negative feedback mechanisms involving PTEN and the serine/threonine phosphorylation of AKT.

Over the past decades, researchers have focused substantial attention on the PI3K/Akt signaling pathway, which is a key intracellular signaling pathway implicated in a range of essential cellular activities, and its physiological functions have progressively come to light. In neurons and various other cell types, PI3K and Akt are involved in key processes, particularly crucial within the central nervous system. Specifically, as upstream modulators such as insulin, growth factors, and cytokines activate the pathway, activated PI3K promotes intracellular production of second messengers PI(3,4)P2 and PIP3, which cause intracellular phosphatidylinositol-dependent kinase-l (PDK1) and inactive Akt to move to the inner surface of the plasma membrane (Jiang et al., 2022). PDK1 can phosphorylate and activate Akt, which subsequently inhibits its downstream molecule GSK3 (Figure 2). A large body of evidence supports the multiple roles of the PI3K/Akt signaling pathway in regulating oxidative stress (Jiao et al., 2022), autophagy (Zhang et al., 2023), inflammatory response (Meng et al., 2021), cell growth (Huang et al., 2021), cell differentiation (Liu F. et al., 2022), and cell cycle (Yuan et al., 2020). A wealth of evidence has confirmed the substantial involvement of all essential components of the PI3K/Akt pathway in the pathogenesis of AD. This review primarily focuses on recent studies regarding the functions of the PI3K/Akt signaling pathway in AD, as well as the role of pharmaceuticals and herbal remedies that influence this pathway. The figures in this manuscript were created using Adobe Illustrator 2020 (Adobe Systems Incorporated, San Jose, CA, United States).

## 2 The role of PI3K signaling pathway in the pathogenesis of AD

### 2.1 Different stages of AD and the selection of mouse model

**Figure 3:**
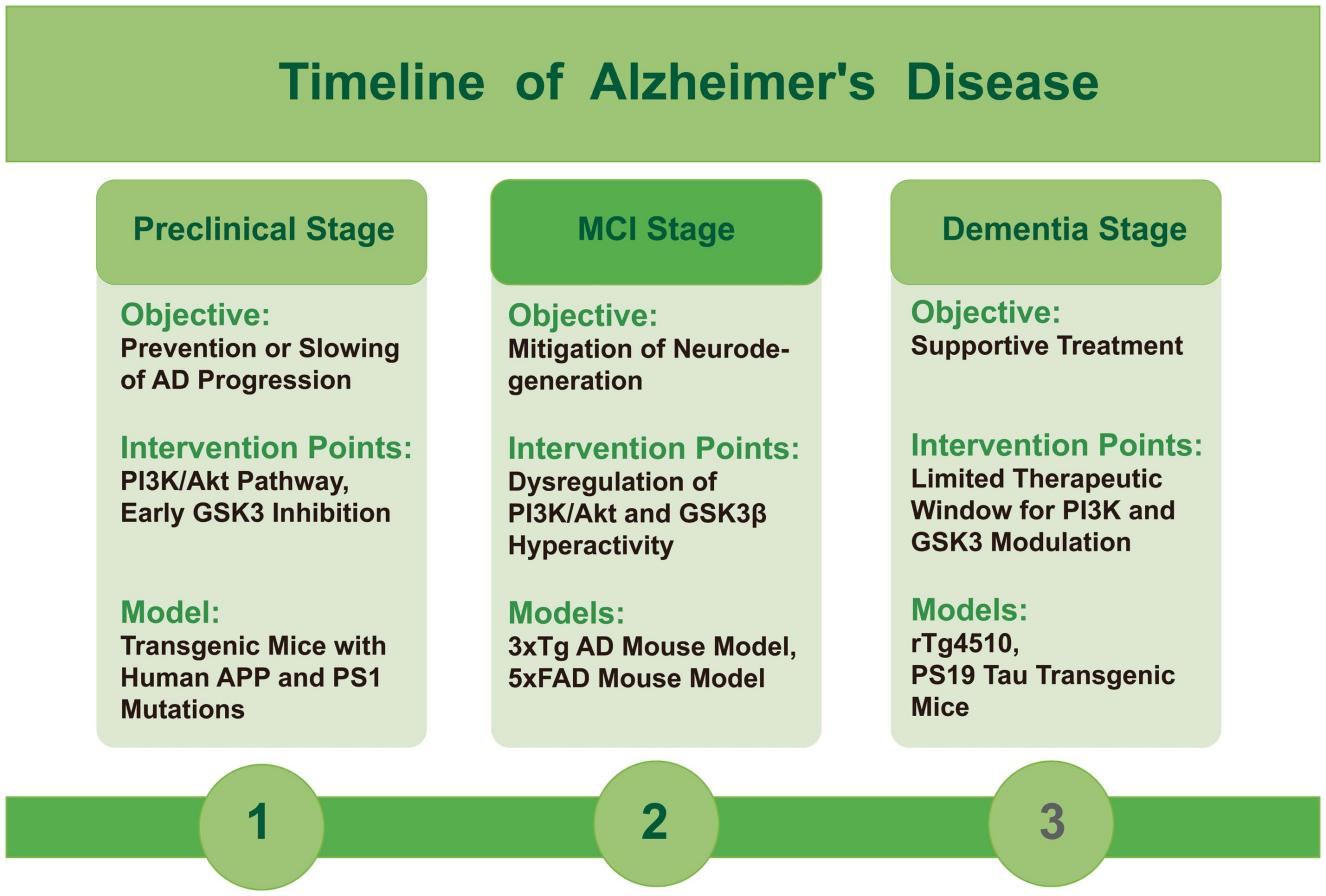
Timeline figure of Alzheimer’s disease, indicating the role of PI3K and GSK3 and mice models in different stages.

In AD, the functions of PI3K and GSK3 vary by stage, underscoring the importance of stage-specific mouse models for research. Here, we describe the progression of AD through its different stages, along with the corresponding mouse models that reflect these stages (Figure 3). During the preclinical stage of AD, an intervention targeting the PI3K/Akt pathway and GSK3 early in the disease may help prevent or slow the advance of the disease. This strategy could be beneficial in the early and mild cognitive impairment stages when the pathological hallmarks of AD are initially emerging (Razani et al., 2021). During this stage, selecting mouse models that can simulate the initial deposition of Aβ and early phosphorylation of tau protein is crucial. For instance, transgenic mice carrying human APP and PS1 mutations typically exhibit Aβ deposition at an early age, reflecting the early pathological changes in AD (Yokoyama et al., 2022).

In the mild cognitive impairment (MCI) stage, as the disease progresses, the dysregulation of the PI3K/Akt signaling axis and the consequent hyperactivity of GSK3β contribute to the formation of toxic tau species and the accumulation of Aβ. Modulating these pathways at this stage may help to mitigate the ongoing neurodegeneration. During this stage, it is beneficial to choose mouse models that display pronounced Aβ plaques, NFTs, and cognitive decline. The triple-transgenic (3xTg) and 5xFAD mouse models are typical for this stage, showing a range of AD features such as plaque formation and abnormal tau protein (Götz et al., 2018).

In the dementia stage, the therapeutic window for modulating PI3K and GSK3 may be more limited, with treatment primarily focused on supportive care. While some studies suggest potential benefits of targeting these pathways, the overall effectiveness may be reduced due to the advanced neuronal damage and extensive plaque and tangle formation (Lauretti et al., 2020). During this stage, the selection should focus on mouse models that exhibit severe neurodegeneration and significant loss of cognitive function. Models such as the rTg4510 and PS19 tau transgenic mice, which express mutant tau protein, effectively simulate the formation of NFTs and associated neurodegeneration, aligning with the late-stage characteristics of AD (Jankowsky and Zheng, 2017).

### 2.2 PI3K/Akt signaling pathway and tau

As a microtubule-associated protein (MAP), Tau protein is enriched in axons and acts as a neurite microtubule stabilizer, regulating tubulin polymerization (Gabbouj et al., 2019). The two most common pathological indicators of AD related to tau pathology are hyperphosphorylated Tau and NFTs. AD etiology is significantly influenced by tau hyperphosphorylation, mainly at serine (Ser) or threonine (Thr), so molecules that reduce phospho-tau accumulation may represent an effective treatment strategy (Bloom, 2014). The degree of tau phosphorylation significantly influences its function and affinity for microtubules. Tau has over 85 different phospho-sites and its dephosphorylated form promotes microtubule assembly. However, phosphorylation reduces tau’s affinity for tubulin, leading to its detachment from the cytoskeleton and accumulation in the cytoplasm. This disruption of microtubules and loss of neuronal integrity ultimately result in NFT formation. Among more than three amino acid residues involved in abnormal tau phosphorylation, the phosphorylation of Thr-231 and Ser-396 is particularly responsible for enhanced NFT production (Rajput et al., 2020). Recent reports have shown that Aβ does not function as the only upstream molecule of tau, and Aβ removal alone is insufficient to reduce pathological tau deposition, suggesting that additional factors beyond amyloid contribute to downstream tau accumulation. In a word, tau pathology may occur independently of Aβ deposition, and studies targeting Aβ and tau should be performed simultaneously (Avgerinos et al., 2021; Oddo et al., 2003).

The extent of tau phosphorylation at specific sites is predominantly regulated by certain protein kinases and phosphatases. Notably, GSK3 and cyclin-dependent kinase 5 (CDK5) have been identified as key protein kinases involved in tau hyperphosphorylation. Extensive data demonstrates that GSK3 plays a significant role in AD pathogenesis, impacting tau hyperphosphorylation, memory loss, intracellular A*β* accumulation, and inflammatory responses (Drewes et al., 1995; Wadhwa et al., 2020). The activation of GSK3 is not only intimately connected to the characteristic clinical alterations of both familial and sporadic AD, but it is also directly linked to cognitive impairment in the absence of AD pathology (King et al., 2014).

As the key kinase for tau protein hyperphosphorylation, GSK3 induces cytoskeletal instability, promotes tau aggregation, and leads to neuronal death. The subunits *α* and β are two isoforms of GSK3 in mammals, and each isoform, with ubiquitous distribution among cell types and tissues, is encoded by a separate gene and acts on different substrates (Soutar et al., 2010). It has been found that the catalytic domains of these two isoforms share similar biochemical characteristics and exhibit 98% amino acid sequence identity (Wadhwa et al., 2020). As a constitutively activated kinase, GSK3β was the first tau-related serine/threonine kinase to be identified, highly expressed in neuronal tissue, especially in the hippocampus, with its expression level increasing with age (Lee et al., 2006). Furthermore, GSK3 is regulated primarily by the inhibition of serine phosphorylation, a process mediated by different Ser/Thr kinases. In particular, phosphorylation at Ser-21 inhibits GSK3α, whereas phosphorylation at Ser-9 (Stambolic and Woodgett, 1994) inhibits GSK3β and phosphorylation at Tyr216 activates it (Jope et al., 2007; Wang et al., 2018). For GSK3β, there are two splice variants (Lin J. et al., 2020). At many AD-related sites, including Thr-181, Ser-199, Ser-202, Thr-205, Thr-212, Ser-214, Thr-217, Thr-231, Ser-396, and Ser-404, tau is phosphorylated by the active form of GSK3β. In AD brains, GSK3β also co-localizes with NFT (Liu et al., 2003; Tan et al., 2018).

Stimulation of the Wnt or insulin signaling pathways, which are negatively regulated by GSK3, has been observed to inhibit tau phosphorylation (Hoeflich et al., 2000). Upon stimulation by insulin, downstream target protein PI3K activity is elevated, subsequently leading to the activation of PDK1. AKT is activated when PDK1 phosphorylates PIP2 to PIP3. GSK3 is subsequently inhibited through the phosphorylation of serine 21 for GSK3α or serine 9 for GSK3*β* (Salkovic-Petrisic et al., 2006).

The Wnt signaling transduction cascade is initiated by Wnt, the evolutionarily conserved small glycoproteins. A canonical Wnt pathway can also regulate GSK3 activity. Inhibition of GSK3 activity by LiCl, the most prescribed medication for AD, is believed to act by emulating Wnt signaling (Ozbek et al., 2007). Deficits in canonical Wnt signaling have been linked to early stages of AD, including synapse loss and dysfunction (Marzo et al., 2016). In addition, defective Wnt signaling can also indirectly affect synapses, a process involving *β*-catenin activation (Folke et al., 2019) and microglial interference (Zheng et al., 2017).

The 19 isoforms of the Wnt protein family, a group of secreted glycoproteins, bind to lipoprotein receptor-related protein (LRP) co-receptors and Frizzled receptors. It is well recognized that Wnt receptor activation results in signal transduction through at least three pathways: a canonical Wnt/*β*-catenin cascade, a non-canonical planar cell polarity pathway, and the Wnt/Ca^2+^ pathway (Palomer et al., 2019). The canonical Wnt pathway links expression of Wnt target genes, such as c-myc, cyclin D1, and Axin2 to cytosolic *β*-catenin, GSK3β, and CK1 (Nusse and Clevers, 2017). In the absence of Wnt signaling, the destruction complex consisting of GSK3α, GSK3*β*, casein kinase-1 (CK1), adenomatous polyposis coli (APC), and Axin breaks down β-catenin. Throughout the process of degradation, the amino-terminal region of *β*-catenin is sequentially phosphorylated by CK1 and GSK3, which enables ubiquitin E3 ligase to recognize it through *β*-transducing repeat-containing proteins (β-TrCP). It contributes to β-catenin ubiquitination and proteasomal breakdown (Tejeda-Muñoz and De Robertis, 2022). However, in the presence of Wnt signaling, the activity of GSK3β is inhibited, and degradation complex activity is suppressed. Therefore, the phosphorylation of β-catenin is also inhibited, thus protecting itself from degradation. Meanwhile, LRP5/6 is recruited to form a complex with the Wnt-bound Frizzled receptor. Subsequently, the Wnt-Fz-LRP6 complex induces the phosphorylation and activation of LRP6. Additionally, the recruitment of the scaffolding protein Disheveled (Dvl) and Axin to the membrane of the LRP signalosome, which contains several GSK3 phosphorylation sites, further reduces GSK3 activity. Dephosphorylated β-catenin increases the transcription of target proteins downstream that are controlled by TCF/LEF when it builds up in the cytoplasm and proceeds to the nucleus (Nusse and Clevers, 2017).

Tau tangles are associated with Wnt/β-catenin signaling, according to recent research. Decreased Wnt signaling generates tau hyperphosphorylation through increased GSK3β activity (Salcedo-Tello et al., 2014), reduction in β-catenin levels, β-catenin-dependent gene expression (Riise et al., 2015), loss of synapses, and memory impairment. Moreover, tau is dephosphorylated by protein phosphatase 2A (PP2A) at several sites with varying degrees of efficiency. The activity of PP2A is markedly reduced in AD brains (Liu et al., 2005), and there was an association between PP2A activity and Wnt signaling in previous studies. In aged rats, PP2A activity decreases with suppression of Wnt signaling, suggesting that tau hyperphosphorylation is caused by the interaction and imbalance between GSK3β and PP2A activity (Salcedo-Tello et al., 2014). Our earlier studies have unambiguously demonstrated how GSK3β and PP2A cooperate to control tau phosphorylation. PP2A methylation is required to promote the dephosphorylation of tau and is modulated by GSK3β through leucine carboxyl methyltransferase 1 and protein phosphatase methylesterase-1. In turn, phosphorylation of GSK3β at Ser-9 is controlled by PP2A. Notably, PP2A and GSK3β dephosphorylate tau with a different preferred order of sites. As previous studies have indicated, the Ser-262/356 site, which is necessary for tau pathology, is PP2A but not the GSK3β site (Alonso et al., 2010). Targeting GSK3β may downregulate PP2A, which would enhance tau phosphorylation at PP2A-sensitive sites. As a result, PP2A is more recommended as a therapeutic target than GSK3β (Wang et al., 2015).

There are two recognized types of AD: the late-onset sporadic form (SAD) and the early-onset familial form (FAD). Mutations in the genes amyloid precursor protein (APP), presenilin-1 (PSEN1), or PSEN2 generate the dominantly inherited type of AD (FAD) (Hardy, 2017). Among them, both GSK3β and tau can co-immunoprecipitate with presenilin. The non-mutated presenilin-1 (PS1) gene can activate the PI3K/Akt signal transduction pathway, which leads to the increase of GSK3 phosphorylation level and inhibits its activity, resulting in the decrease in tau phosphorylation level catalyzed by GSK3, ultimately protecting cells and inhibiting apoptosis (Baki et al., 2004). While the mutant PS1, which is associated with FAD, can bind tightly to GSK3β, which leads to an improved ability of GSK3β to catalyze the phosphorylation of tau, resulting in tau hyperphosphorylation (Liu et al., 2004). Reports state that the non-mutated PS1 that initiates the PI3K/Akt signaling pathway is greatly inhibited by the FAD-related mutant PS1. This, in turn, increases the activity of GSK3, which renders tau abnormally hyperphosphorylated (Haase et al., 2004). This is a potential pathogenesis whereby FAD mutations accelerate disease progression through secondary enhanced GSK3 activity. In human neurons, in addition to affecting tau phosphorylation through effects on GSK3, PS1 mutations also lead to tau accumulation through deregulation of the mammalian target of rapamycin 1 (mTORC1) signaling (Chong et al., 2022).

Several studies indicate that the GSK3β and PI3K/Akt signaling pathways are important targets for the treatment and prevention of AD, as well as for the protection against tauopathy. Triggering receptor expressed on myeloid cells 2 (TREM2) is a transmembrane immunoglobulin superfamily receptor found exclusively on microglia in the central nervous system. Recent studies indicate that TREM2 improves the spatial cognitive capacity of APP/PS1 mice by preventing tau hyperphosphorylation at Ser-199, Ser-396, and Thr-205 and by suppressing neuronal death by activating PI3K/Akt/GSK3β signaling (Peng et al., 2023).

Remarkably, sex hormones are fundamental to regulating the phosphorylation of tau, and the process is connected to both the GSK3β and PI3K/Akt signaling pathways. It is widely recognized that in males, decreased testosterone level increases the risk of AD (Pike, 2017), while in females, decreased plasma testosterone correlates with significantly increased cerebrospinal fluid levels of phosphorylated tau (Sundermann et al., 2020). The function of testosterone in preventing tau hyperphosphorylation has been documented in recent animal studies. In an experiment conducted on 6- and 60-day-old male mice, testosterone was discovered to inhibit the interaction between tau and GSK3β in both cultured hippocampal neurons and neonatal mice, thereby attenuating the tau phosphorylation and cognitive impairment caused by sevoflurane (Yang et al., 2021). In male 3xTg-AD mice, testosterone and its active metabolite, dihydrotestosterone (DHT), significantly decreased phosphorylated tau while increasing the phosphorylation of Akt and GSK3β. This pathway is mediated through the androgen receptor (AR) (Lutshumba et al., 2023). However, while testosterone effectively reduces tau phosphorylation *in vitro*, clinical experimental results remain inconclusive. Clinical studies have shown that lower testosterone levels are linked to greater p-Tau levels in APOE4 carriers. The discrepancy in p-Tau levels between men and women was removed when testosterone was taken into consideration (Sundermann et al., 2020). However, testosterone therapy was reported to fail to improve cognitive function in elderly men (Snyder et al., 2018), which indicates that androgen is only one of the effective factors for neuroprotection, and therapies using testosterone to treat AD remain to be explored.

Estrogen has neuroprotective qualities in an array of *in vitro* and *in vivo* models, which are particularly significant in AD pathophysiology, apart from its essential role played in the reproductive system (Uddin et al., 2020). Previous studies have shown that 17β-Estradiol (E2) inhibited tau hyperphosphorylation by inhibiting the activation of the JNK/c-Jun/Dkk 1 pathway (Zhang et al., 2008). While in C57Bl6/J mice, GSK3β phosphorylation at the serine-9 site is inactivated when the 17β-estradiol pathway is activated, which, in turn, reduces Aβ accumulation as well as hyperphosphorylated tau (Goodenough et al., 2005). Therefore, therapies targeted at estrogen receptors are expected to offer a good clinical application. Belonging to the nuclear receptor superfamily, the estrogen receptor (ER) is a steroid hormone receptor that mediates the biological actions of estrogens and phytoestrogens. Some selective Er*β* receptor agonists, such as gypenoside XVII and genistein, can improve the clinical symptoms of AD through ER-dependent activation of the PI3K/Akt pathway, inducing diminished oxidative stress and decreasing tau phosphorylation (Bagheri et al., 2011; Meng et al., 2014). Furthermore, it has been discovered that p-Tau (Thr-231, Ser-396)/Tau levels were much lower in PC12 cells treated with a combination of Aβ_25-35_ and naringin than in cells treated with Aβ_25-35_ alone (Qiu et al., 2023). These phytoestrogens, or estrogen analogs, produce potent estrogen-like effects and can avoid serious side effects caused by long-term estrogen use, such as breast cancer, uterine cancer, and thrombosis (Rozenberg et al., 2021; Zhang G. Q. et al., 2021; Abou-Ismail et al., 2020).

### 2.3 PI3K/Akt signaling pathway and A*β*

#### 2.3.1 Modulation of Aβ by GSK3

The creation and buildup of extracellular Aβ, derived from the proteolytic processing of APP, serves as the main feature of AD. According to the timing of onset, AD is commonly classified into two basic types: late-onset AD (LOAD) and early-onset AD (EOAD). The former is usually diagnosed before the age of 65 years while the latter develops after the age of 65 years. EOAD is predominantly caused by the overproduction of three rare mutation genes: *APP*, *PSEN1*, and *PSEN2* and is inherited by a dominant autosomal pattern, while the genetics of LOAD are much more complicated (Hardy, 2017). There are three major APP isoforms found in humans due to alternative splicing: APP695, APP751, and APP770, among which APP695 is found exclusively in neurons. There are two pathways to process APP, namely, amyloidogenic and non-amyloidogenic pathways. As the β-secretase enzyme in the former pathway, β-Site APP cleaving enzyme 1 (BACE1) cleaves extracellular domain of the transmembrane APP and releases a soluble APP fragment *β*. Then, *γ*-secretase cleavage occurs, and β- and γ-secretases cleave one another sequentially to generate full-length Aβ aggregates (mostly Aβ_1-40/42_), which are expected to develop into Aβ plaques. Nicastrin (NCT), anterior pharynx-deficient 1 (APH1), presenilin enhancer 2 (PEN-2), and presenilin (PSEN) make up the tetrameric protein complex known as *γ*-secretase (Escamilla-Ayala et al., 2020). In the latter pathway, APP is broken down by the *α*-secretase complex, which is comprised of ADAM-10 and-17 together with the γ-secretase, resulting in a truncated Aβ_17-40/42_ (P3) peptide, or broken down by β-secretase, resulting in a truncated Aβ_1-16_ peptide (Miranda et al., 2021; Khezri et al., 2023; Chow et al., 2010). Moreover, the most neurotoxic subgroups of Aβ_42_ oligomers, specifically the soluble ADDLs (Aβ-derived diffusible ligands), are known for their capacity to permeabilize cellular membranes, leading to cell dysfunction and death, which, in turn, contributes to the cognitive impairment associated with AD (Wen et al., 2018).

PI3K/Akt/GSK3β signaling pathway has regulatory effects on Aβ. Chiang et al. illustrated that decreased PI3K activity can reverse Aβ_42_-mediated learning impairment in transgenic fly lines (Chiang et al., 2010). Investigators have also shown that overexpression of Akt significantly attenuated Aβ_1-42_-induced apoptosis, and its protective mechanisms include the activation of caspase-3 and JNK, downregulation of Bad and Bax, and upregulation of Bcl-xL and Bcl-w (Yin et al., 2011). In addition, Aβ_1-42_ was reported to increase Akt phosphorylation depending on the alpha (7) nicotinic receptor and NMDA receptor (Abbott et al., 2008). In drosophila, reduced Akt activity can mitigate Aβ-induced damage by modulating Notch activity, given that Notch is an intrinsic substrate of γ-secretase (Chen et al., 2019).

Previous studies suggested that the APP and PS1 are substrates of GSK3β, and during Aβ production, GSK3β plays a role by affecting β-secretase (Lauretti et al., 2020). BACE1 gene expression is activated by GSK3β, leading to increased β-secretase processing of APP and Aβ generation. Furthermore, GSK3β suppresses the γ-secretase complex and reduces α-secretase via PKC and ADAMs, which function as GSK3β substrates and ultimately affect Aβ synthesis (Saha et al., 2021; Pal et al., 2021). In 5 × FAD mice co-expressing mutant APP and presenilin-1, increased expression of GSK3 isoforms impedes Aβ clearance, increasing Aβ plaque formation and memory impairments. While L803-mts, the substrate-competitive GSK3 inhibitor restores lysosomal acidification, allowing Aβ to be cleared and mTOR to be reactivated (Avrahami et al., 2013). On the flip side, Aβ aggregation can induce GSK3β activity through Aβ-mediated neuroinflammation and oxidative stress; it can also increase GSK3β activity by preventing PI3K activation and subsequently preventing Akt activation (Townsend et al., 2007). In addition, specific Aβ antibodies have been shown to reduce GSK3β activity, suggesting an interaction between GSK3β and Aβ-peptide (Ma et al., 2006). However, considering the intricate mechanisms involved in Aβ formation and clearance, inhibiting GSK3β alone has limited effects on reducing the Aβ level. Additionally, as GSK3 activation results in tau hyperphosphorylation and neurofibrillary lesions, we propose that GSK3 could serve as a mediator in the interplay between the pathophysiology of NFT and amyloid plaques.

#### 2.3.2 Modulation of Aβ by oxidative stress

Oxidative stress is well recognized as a contributor to the aging process and the development of a variety of neurological illnesses. It interacts with Aβ accumulation, resulting in a defect of mitochondrial respiratory complexes and eNOS deficiency, and is regulated by the PI3K/Akt pathway (Magrané et al., 2005). Metal ions in Aβ are crucial during oxidative stress. The accumulation of metal ions is among the main contributors to oxidative stress, including zinc, iron, and copper in Aβ, which can directly bind Aβ and encourage its aggregation. This is one of the characteristics of AD (Cheignon et al., 2018). Disruption of Fe and Cu homeostasis can lead to elevated levels of free or loosely bound ions, which may be reduced to Cu (I) or Fe(II) by biological reductants such as ascorbic acid or intracellular glutathione. Following a reaction with hydrogen peroxide or dioxygen, these compounds produce superoxide and hydroxyl radicals, respectively (Halliwell, 2006; Faller and Hureau, 2012).

Mitochondrial damage is an important pathway causing oxidative stress, leading to ROS surges including superoxide radical anion, hydrogen peroxide, and hydroxyl radical, which further increase mitochondrial respiratory chain damage. This process occurs early in AD, predating AD clinical symptoms and Aβ pathology (Tönnies and Trushina, 2017). Notably, the PI3K pathway affects mitochondrial injury through multiple pathways. When p-Akt (phosphorylated Akt) expression is significantly decreased, insulin fails to induce expression of GLUTs (glucose transporter) and promote glucose uptake, thereby disrupting glucose metabolism and consequently impairing mitochondrial aerobic respiration, which, in turn, augments ROS production. Furthermore, mitochondrial function affects Aβ processing. Studies have shown that oxidative stress caused by hydrogen peroxide raises the amount of A*β* deposited in brain arteries through the amyloidogenic pathway, potentially associating with the phosphorylation of p42/44 MAPK (Muche et al., 2017). As a result, Aβ oligomers that build up in the inner membrane or matrix reduce the respiratory chain complexes III and IV’s enzymatic activity (Caruso et al., 2019). Enzymes I, IV, and V of the mitochondrial complex exhibit increased activity when Aβ is injected into lateral ventricles using a bilateral intracerebroventricular route, as demonstrated by Garabadu and Verma (2019). However, this impact is considerably decreased by Exendin-4 through a PI3K/Akt-mediated mechanism.

Mitochondrial dysfunction not only causes increased ROS but also increases reactive nitrogen species (RNS), including nitric oxide (NO), and peroxynitrite (ONOO-) (Singh and Devasahayam, 2020). Previous research has indicated that eNOS(−/−) mice exhibit elevated levels of BACE1 activity and Aβ_40_ plaques compared to late middle-aged wild-type mice (Austin et al., 2013). Whereas recent research showed that compared with complete eNOS deficient (eNOS−/−) mice, APP/PS1/eNOS+/− mice showed more severe spatial working memory deficits. In fact, partial eNOS impairment contributes to an even higher rise in Aβ plaque in the brain by upregulating BACE1, downregulating insulin-degrading enzymes, and increasing immunoreactivity and microglial expression (Austin and Katusic, 2020; Linjuan et al., 2024).

Given that ENOS is downstream in the PI3K-Akt pathway, diseases stemming from ENOS damage can benefit from targeting this signaling system therapeutically. According to reports, exogenous hydrogen sulfide can activate the PI3K/Akt/eNOS pathway, protecting endothelial cells from damage caused by excessive hyperglycemia (Lin F. et al., 2020), and sulforaphane regulates eNOS activation via PI3K/Akt signaling in human endothelial cell. Furthermore, dietary supplements like as L-arginine and L-citrulline have been shown to be effective in enhancing nitric oxide production, suggesting that food therapy is a viable and essential alternative that warrants consideration in the control and avoidance of AD (Kiani et al., 2022).

#### 2.3.3 Modulation of Aβ by autophagy

The preservation of neuronal morphology and homeostasis largely relies on autophagy, a prosurvival mechanism. Additionally, pathways involved in the degradation and clearance of abnormal protein products in cells also include the ubiquitin–proteasome system (UPS), microglial activation, and β-amyloid_1–42_-degrading enzyme releasing (Wang and Le, 2019). Through the equilibrium of these pathways, neuronal cells remove abnormal protein aggregates and maintain protein homeostasis (Yang and Zhang, 2023). The autophagy pathway involves proteins or organelles being transported to lysosomes for degradation via autophagosomes to form autolysosomes. An autophagosome develops in a distant axon of a neuron before moving retrogradely to merge with a lysosome in the soma. However, due to the anatomical characteristics of neurons, which feature highly polarized axons and dendritic compartments, the length of axons is much longer than that of the soma, and lysosomes are rarely distributed in distal axons. As a result, axonal autophagosomes must be transported over a long distance, which makes the autophagic function of neurons easily impaired, thus involved in various proteinopathies (Cason et al., 2022), including AD (Zhang Z. et al., 2021), Huntington’s disease (Kim et al., 2021), and Parkinson’s disease (Themistokleous et al., 2023). Premature autophagic vacuoles (AVs), which accumulate in dystrophic neuritis, have been seen in postmortem AD brains of animal models and patients. A substantial body of research has indicated that autophagy dysregulation can be seen in AD brains, particularly early in the pathogenesis (Li et al., 2017). Moreover, several investigations have demonstrated that the dysfunction of the autophagy–lysosome pathway may occur before the formation of NFTs or Aβ plaques (Ntsapi et al., 2018).

There are at least three well-recognized types of autophagy, known as chaperone-mediated autophagy (CMA), microautophagy, and macroautophagy, among which macroautophagy is considered as the major type and most associated with AD. As macroautophagy is activated, the unc-51-like kinase (ULK) and vacuolar protein-sorting 34 (VPS34) complexes initiate autophagosome formation, and the kinase activity of ULK promotes local production of PI3P (Zhang Z. et al., 2021). Meanwhile, the core subunit of the VPS34 complex, Beclin-1, decreases as AD progresses, and PI3P synthesis, catalyzed by the VPS34 complex, is downregulated in a similar way in AD brains (Yuan et al., 2022). In particular, abnormalities in autophagy lead to elevated *γ*-secretase activity, exacerbating Aβ buildup in the brain and causing amyloid processing of APP.

The primary downstream target of PI3K-Akt signaling, mTOR, is responsible for the control of autophagy. As a serine/threonine kinase, mTOR controls transcription, protein synthesis, cell division, growth, and autophagy. In particular, mTOR can consolidate and sustain memory function by boosting both synaptic plasticity and protein synthesis in dendrites and synapses through the formation of two multiprotein complexes, mTORC1 and mTORC2 (Querfurth and Lee, 2021). In mouse AD models, synaptic density and plasticity can be restored by using Akt or PI3K activators to stimulate the insulin/mTOR pathway (Yi et al., 2018). Additionally, TORC1 is a crucial negative regulator of autophagy, meaning that autophagy is induced by negative regulation of mTORC1 and suppressed by activated mTORC1. Recent evidence suggests that mTORC1 inhibition, both *in vivo* and *in vitro*, can reduce amyloid secretion via neuronal autophagy induction (Benito-Cuesta et al., 2021). On the one hand, dysregulated mTOR activation can exacerbate Aβ generation and deposition, either by inhibiting Aβ clearance or by increasing Aβ generation via pathways such as insulin resistance (Luchsinger, 2008). On the other hand, injection of naturally secreted Aβ into the rat brain has been reported to increase mTOR signaling and activity (Davoody et al., 2024).

PTEN, a lipid phosphatase and protein tyrosine phosphatase, dephosphorylates and changes PI3K-generated PIP3 into PIP2, inhibiting the PI3K/Akt/mTOR signaling pathway (Hodges et al., 2018). Research has indicated that the ionophore alborixin can block the PI3K/Akt pathway by binding to PTEN, suppressing mTOR, which subsequently triggers autophagy and eliminates Aβ (Wani et al., 2019). Drugs that directly target proteins associated with the mTOR signaling pathway confer an immense potential for application in AD treatment. However, given the role of mTOR pathway in several vital biological processes, there is a risk of toxicity for patients due to the prolonged inhibition of mTOR by drugs such as rapamycin. Therefore, finding targets for new AD drugs that are more precise than existing drugs is needed, such as p70S6K1/2 and other mTOR-dependent effectors (Caccamo et al., 2015).

## 3 The therapeutic possibility of the modulation of PI3K/Akt/GSK3β in AD

Drug treatment is an important option for AD patients; however, only a few medications are authorized for use in therapeutic settings, such as galantamine, memantine, donepezil, rivastigmine, and the Chinese herb extract huperzine A. Given the function of the PI3K-Akt pathway in AD, several synthetic and natural modulators, ranging from chemicals to herbs, have been shown to alleviate the symptoms of this degenerative condition in various *in vivo* and *in vitro* models. Among these modulators, natural products can be classified into saponins, phenylpropanoids, flavonoids, non-flavonoid polyphenols, and others. Many of these compounds are derived from traditional Chinese medicine, offering increased safety and reduced toxicity in therapeutic applications, thus holding significant potential as future drug candidates. When comparing natural herbal inhibitors with commercially available GSK3B inhibitors, it is important to consider their origin and accessibility, with natural herbal inhibitors often being more readily available, especially in regions where traditional medicine is prevalent. They are known for their multi-target action, which could potentially overcome drug resistance in cancer cells, in contrast to the targeted, single-focus approach of commercial inhibitors (Yoshino and Ishioka, 2015). Although natural herbal inhibitors are generally perceived as having fewer side effects due to their holistic approach and historical use, they may lack the stringent safety assessments that accompany the development of commercial inhibitors, which are often synthetic and could have unforeseen side effects. In terms of clinical research and evidence base, commercial inhibitors are typically backed by extensive studies validating their safety and efficacy, whereas natural herbal inhibitors, despite their long use in traditional medicine, often lack this level of scientific validation and clinical trial support (Thapa et al., 2023).

However, the action mechanism, molecular targets, and, most importantly, clinical efficiency of many novel modulators remain to be further studied. In the following part of this article, some important modulators of PI3K/Akt/GSK3β are discussed, and Table 1 offers a summary for readers of the experimental cell, animals, dosage, and duration utilized in studies using natural and synthetic drugs.

**Table 1:** List of herbal and natural compounds applied for the treatment of AD.

Compounds	*In vitro*/vivo models/human trial	Treatment dose	Treatment time	Mechanism	References
Ginsenoside Rg1	Male Kunming mice	10,20 mg/kg	20 days	Restore the reduction in FGF2 and BDNF and restore TrkB/Akt pathways to limit apoptosis.	Zhong et al. (2020)
Ginsenoside Rg2	Male Sprague–Dawley rats	25, 50, 100 mg/kg/day	6 weeks	Elevate the ratio of Bcl-2/Bax, reduce caspase-3 cleavage, and boost Akt phosphorylation.	Cui et al. (2021)
Salidroside (Sal)	Cortical neurons of embryonic day 18 C57BL mice; Transgenic *Drosophila* models	50, 100, or 200 μM for cortical neurons; 2, 6, and 20 μM for *Drosophila*	24 h for cortical neurons; 30 days for *Drosophila*	Elevate p-Akt and reinstate p-mTOR and p-p70S6K expression.	Zhang et al. (2016)
	APP/PS1 mice	0.3 mg/mL	2 months to 12 months	Reduce Aβ levels, upregulate phosphatidylinositide PI3K/Akt/mTOR signaling, and express PSD95, NMDAR1, and calmodulin-dependent protein kinase II.	Wang et al. (2020)
Oxyphylla A	N2a/APP cells, SAMP8 mice model of AD	Concentration of 12.5,25,50,100 μM for cell; 10 mg/kg,20 mg/kg for mice	24 h for cell, 6 weeks for mice	Turn on the Nrf2/Keap1/HO-1 and Akt/GSK3b pathways to reduce Aβ.	Bian et al. (2021)
Scutellaria baicalensis Georgi stems and leaves flavonoids (SSF)	Model of male Wistar rats of AD induced by intracerebroventricular injection of Aβ_25-35_ in combination with AlCl3 and RHTGF-β1	35, 70, and 140 mg/kg	37 days	Overexpress TRKB, PI3K, Akt, CREB, and IGF2.	Liu Q. Q. et al. (2022)
Cinnamaldehyde (Cin)	Sporadic AD rat model	100 mg/kg(intraperitoneal)	2 weeks	Reduce the amount of phosphorylated Akt in the hippocampus and raise the phosphorylation of GSK3β at Ser-9.	Bagheri-Mohammadi et al. (2022)
Licochalcone A (LicA)	SH-SY5Y cell	0, 10, 20, 50, 100 μM	24 h	Inhibit autophagy, activate PI3K/Akt/mTOR signaling, increase the level of Bcl-2, and decrease the level of BAX.	Guo et al. (2022)
Galangin	PC12 cells of OA-induced neurotoxicity	0.25, 0.50, 0.75, and 1.00 μg/mL	48 h	Increase the vitality of cells treated with OA, lower the levels of β-secretase, Aβ_42_, and p-tau, stifle the expression of Beclin-1 and p-GSK3β, and encourage the p-Akt and p-mTOR expression.	Huang et al. (2019)
Mori Fructus ethanol extract (ME)	Male ICR mice	20 mg/kg, 100 mg/kg, and 500 mg/kg	2 weeks	Reduce tau phosphorylation, increase Akt and GSK3β phosphorylation, and lessen Aβ-induced cell damage	Kim et al. (2015)
Fructus broussonetiae (FB)	APP/PS1 mice	200 μL of FB (0.1, 0.15, and 0.3 g/mL)	2 months	Reduce cell apoptotic signals while increasing β-catenin and AKT signaling	Li Y. H. et al. (2021)
Asiatic acid	PC12 cells treated with 20 μM Aβ_25-35_	5, 10 or 20 μM	24 h	Activate GSK3β and Akt phosphorylation, inhibit IκBα degradation, and prevent p65 nuclear translocation.	Cheng et al. (2018)
Schizandrol A (SchA)	Differentiated SH-SY5Y cells; primary hippocampal neurons	2 μg/mL	24 h	Reduce p62, inhibit the rise in LC3-II/LC3-I, and lessen the inactivation of the PI3K/Akt/mTOR pathway.	Song et al. (2020)
Schisandrin (SCH) and nootkatone (NKT)	Aβ1-42_−_induced PC12 cells	SCH (50 μM) and NKT (10 μM)	20 h	Lower the levels of NF-κB, IKK, IL-1β, IL-6, TNF-α, cleaved caspase-3, and LC3-II.	Qi et al. (2020)
*Gardenia jasminoides* J. Ellis extract GJ-4	APP/PS1 transgenic mice	10, 20, and 50 mg/kg	12 weeks	Reduce neuroinflammatory reactions, lower the amount of Aβ, and reduce Tau phosphorylation at specific sites: Ser-396, Thr-231, and Ser-202/Thr-205.	Zang et al. (2021)
Sulforaphene (SF)	Adult male SD rats	25 and 50 mg/kg	6 weeks	Reduce tau protein phosphorylation at the Thr-205, Ser-396, and Ser-404 sites; increase the p-Akt (Ser-473)/Akt and p-GSK3β (Ser-9)/GSK3β ratios.	Yang et al. (2020)
Shenzhiling oral liquid (SZL)	Aβ_42_-induced oligodendrocyte OLN-93 cells; APP/PS1 mice	Concentration of 4, 8, 16, and 20% for cell; 2.9 mL/kg/d for mice	24 h for cell; 3 months for mice	Elevate MBP, PLP, MAG, p-PI3K, PI3K, p-Akt, Akt, p-mTOR, and mTOR expression in the APP/PS1 mice and Aβ_42_-induced OLN-93 cell hippocampi.	Zheng et al. (2021)
Chaihu Shugan San (CSS)	Differentiated PC12 cells induced by Aβ	250, 500 μg/mL	24 h	Reduce the production of Bax and prevent Aβ from inhibiting Akt phosphorylation.	Zeng et al. (2019)
Exendin-4	Adult male Wistar albino rats received stereotaxic injection of Aβ	5 μg/kg, i. p	14 days	Increase Aβ-induced reduction in phosphorylated Akt levels, respiratory control rate, ADP/O, and mitochondrial integrity	Garabadu and Verma (2019)
Resveratrol–selenium–peptide Nanocomposites	AlCl3 and D-gal-induced mice model of AD	50 mg/kg	16 weeks	Reduce Aβ-induced neural inflammation by means of the nuclear factor kappa B/mitogen-activated protein kinase/Akt signal transduction pathway.	Li C. et al. (2021)
Magnesium-L-threonate (MgT)	Aβ_25-35_-induced HT22 cells; APP/PS1 transgenic mice	50 μmol/L for cell; 910 mg/kg/d for mice	12 h for cell; 3 months for mice	Reduce HIF-1α and Bax expression, increase Bcl-2 expression, initiate the PI3K/Akt pathway, and improve cognitive impairment.	Xiong et al. (2022)
Sodium orthovanadate (SOV)	ICV-STZ-induced rats	5 mg/kg, 10 mg/kg	21 days	Increase the expression of the genes for IR, IRS-1, PI3K, and AKT and decrease the expression of GSK3β	Akhtar et al. (2020)

Ginsenoside Rd, belonging to saponins, is the active compound in *Panax ginseng* C. A. Meyer, which is famous for its effects to reduce neurological damage in neurodegenerative diseases through several pathways, including anti-inflammatory, antioxidant, and anti-apoptosis (Chen Y. Y. et al., 2022). Researchers have demonstrated that Ginsenoside Rd. acts through estrogen receptor *α* (ERα) to affect the MAPK/ERK and PI3K/Akt pathways, increasing sAPPα levels and decreasing extracellular Aβ in OVX rats (Yan et al., 2017). According to research on aging mice given D-galactose and AlCl3, ginsenoside Rg1 reverts the aging-induced decrease in FGF2 and BDNF, stimulates the TrkB/Akt pathways in the prefrontal cortex and hippocampus, and ultimately prevents apoptosis (Zhong et al., 2020). Yang et al. also demonstrated how Rg1 modulates the GSK3β/Wnt signaling pathway to decrease Tau protein phosphorylation, Aβ_1-42_ deposition, and BACE1 expression in AD tree shrews (Yang et al., 2022). Additional research has verified that ginsenoside Rg2 reduces cognitive impairment caused by Aβ_25-35_, raises the Bcl-2/Bax ratio, and prevents the rat model of caspase-3 cleavage by initiating the PI3K/Akt pathway (Cui et al., 2021). Regarding preclinical and clinical trials, existing evidence focused on combination treatment and dietary supplements of ginseng or red ginseng as a whole, instead of its specific components, indicating that more high-quality clinical trials remain to be conducted (Li J. et al., 2021).

Salidroside (Sal) is a bioactive compound derived from *Rhodiola rosea* L, a valuable medicinal plant. It was reported to exhibit neuroprotective activities, notably against AD, through a range of mechanisms including antioxidant defense, apoptosis regulation, and modulation of amyloid-beta levels via the PI3K/Akt/mTOR pathway. Additionally, it enhances cognitive function by affecting p-GSK3β and p-tau, inhibits neuroinflammation and apoptosis via the SIRT1/NF-κB pathway, protects against excitotoxicity, and regulates amyloid precursor protein processing (Zhong et al., 2018). In particular, Sal has not yet shown any detectable toxicity or deleterious effects in acute trials or other toxicity investigations (Zhang X. et al., 2021). According to earlier studies, Sal works as a novel therapeutic drug for cardiovascular diseases by inhibiting platelet activity via the Akt/GSK3β signaling pathway (Wei et al., 2020). According to research by Zhu et al. on the neuroprotective benefits of sal for aging models, Sal increases the production of the telomerase reverse transcriptase (TERT) protein through the PI3K/Akt pathway. The TERT protein, as the catalytic subunit of telomerase, plays a crucial role in maintaining telomere length, which is inherently linked to cellular aging. By increasing TERT expression, salidroside helps to slow the rate of telomere shortening, thereby delaying the cellular aging process (Zhu et al., 2021). These results align with similar studies in the AD model. Sal may enhance PC12 cell survival and proliferation by activating the ERK1/2 and Akt signaling pathways, according to prior *in vitro* research (Liao et al., 2019). Additionally, the researchers showed that Sal raises the expression of PSD95, NMDAR1, and calmodulin-dependent protein kinase II and reduces the levels of both soluble and insoluble Aβ via upregulating PI3K/Akt/mTOR signaling in an experiment to assess its neuroprotective effects on APP/PS1 mice (Wang et al., 2020). In a study on the *Drosophila* model, the results are similar to Zhang et al. (2016). In particular, natural Sal is present in very low concentrations, necessitating the use of synthesized products as backups in clinical settings (Zhang X. et al., 2021).

Curcumin, belonging to flavonoids, is the major compound of turmeric, which is an herb native to southern Asia, mainly extracted from the rhizome of *Curcuma longa*. Curcumin has been extensively examined for its remarkable capacity to alter AD pathogenesis through multiple mechanisms, including reducing the formation and neurotoxicity of Aβ and tau. However, the implication of the PI3K/Akt/GSK3β pathway in these processes has not been illustrated in detail (Tang and Taghibiglou, 2017; Reddy et al., 2018). Recent reviews pointed out that two significant signaling pathways, PI3K/Akt/GSK3 and PI3K/Akt/CREB/BDNF, mediate the main mechanisms of curcumin neuroprotection, namely antioxidant, anti-inflammatory, and anti-apoptotic functions (Kandezi et al., 2020). Researchers have demonstrated that curcumin improves memory recall impairment in scopolamine-induced mouse models by restoring Akt and GSK dephosphorylation (SoukhakLari et al., 2018). Wang et al. have shown how exosomes produced from cells primed with curcumin prevent Tau protein phosphorylation by triggering the Akt/GSK3*β* pathway, which alleviates symptoms in the AD rat model. It is widely recognized that the low bioavailability of curcumin hinders its therapeutic effects, while exo-cur boasts highly effective BBB-crossing, offering a solution to this problem (Wang et al., 2019). Vorinostat, also known as suberoylanilide hydroxamic acid (SAHA), is an FDA-approved histone deacetylase inhibitor. Silibinin, a naturally occurring flavonolignan, has been recognized for its hepatoprotective and antioxidant properties. Studies focused on combination therapy, revealing that curcumin, vorinostat, and silibinin together alter the Akt/MDM2/p53 pathway, shielding PC12 cells from the toxicity caused by Aβ_25–35_ (Meng et al., 2019). Recent clinical trials appear to be positive as well. Dietary supplementation with curcumin has shown that it reduces circulating levels of islet amyloid polypeptide (IAPP) and GSK3*β*, significantly alleviating insulin resistance. IAPP is a hormone that, when misfolded, forms toxic amyloid deposits in pancreatic islets, contributing to beta cell dysfunction and a key pathological feature of type 2 diabetes (Thota et al., 2020). However, the reduction in circulating levels of risk molecules is still far from the improvement of clinical symptoms and more convincing evidence is required.

Oxyphylla A is a recently identified compound extracted from Alpinia oxyphylla, a plant employed historically to treat brain-related disorders and also referred to as Yi Zhi in Chinese herbal medicine. Bian et al. demonstrated how oxyphylla A acts as a neuroprotective agent in N2a/APP cells and SAMP8 mice by lowering the expression levels of APP, Aβ_1-40_, and Aβ_1-42_ and inhibiting oxidative stress through Nrf2 activation via the Akt/GSK3β pathway (Bian et al., 2021). Scutellaria baicalensis Georgi stems and leaves flavonoids (SSF) is a component with multi-pharmacological neuroprotective activities, extracted from the stems and leaves of Scutellaria baicalensis Georgi. A previous *in vivo* investigation found that by upregulating the PI3K-Akt-CREB signaling pathway and the mRNA and protein expressions of TRKB, PI3K, Akt, CREB, and IGF2, SSF may enhance cognitive impairment and neurogenesis in AD model rats (Liu Q. Q. et al., 2022). Recent investigations have indicated that the main ingredient in cinnamon, cinnamaldehyde (Cin), has dose-dependent neuroprotective properties. Recent research indicates that Cin selectively triggers caspase-3 activation, hippocampal IRS-1/Akt/GSK3β signaling disruption, and Aβ aggregation. This results in improved AD symptoms in the sporadic AD rat model, which is comparable to insulin (3 mU/ICV) therapy (Bagheri-Mohammadi et al., 2022). Licochalcone A (LicA), as a dietary and herbal flavonoid extracted from the root of *Glycyrrhiza glabra*, was reported to have quite a few biological activities, including protective effect against Aβ peptide. In the experiment to test the neuroprotective effects of Sal on SH-SY5Y cells, Guo et al. illustrated that LicA significantly reduces Aβ_25-35_-induced autophagy and elevates anti-apoptotic protein levels along with PI3K, Akt, and mTOR activation. Galangin, as a natural flavonol that is an important active component of *Alpinia officinarum*, has been considered useful in treating or delaying AD. Galangin has been shown to decrease β-secretase and acetylated H3 in the promoter regions of β-secretase in SH-SY5Y cells (Zeng et al., 2015). Furthermore, Huang et al. showed that in okadaic acid-induced PC12 cells, galanin could suppress autophagy through the Akt/GSK3β/mTOR pathway by raising p-Akt and p-mTOR and decreasing p-GSK3β levels (Huang et al., 2019).

Mori Fructus (*Morus alba* L. fruit, mulberry) was believed to exert neuroprotective effects in several models in recent years, including AD. Researchers have examined the neuroprotective qualities of Mori Fructus ethanol extract (ME) in rat primary hippocampus cells in response to Aβ-induced toxicity. ME prevented Aβ_25–35_-induced cell damage both *in vitro* and *in vivo*. This protective effect was mediated by activation of GSK3β and Akt and results in the reduction in tau phosphorylation and apoptosis in mouse models induced by the toxicity of Aβ_25–35_ (Kim et al., 2015). In addition, pharmacological and clinical studies suggest Chinese herbal medicine Fructus broussonetiae (FB) may be a possible medication for AD therapy. In the experiment to test the neuroprotective effects of FB on APP/PS1 mice, Li et al. demonstrated that FB exerted anti-AD effects both *in vivo* and *in vitro*, and the *in vitro* experiment revealed that cells treated with FB had elevated levels of both AKT and β-catenin signaling (Li Y. H. et al., 2021). Research has indicated that asiatic acid (AA), a naturally occurring pentacyclic triterpene derived from *Centella asiatica*, may aid in lowering the concentration of Aβ in the AD brain. Through regulating PI3K/Akt/GSK3β signaling, AA has been shown to protect differentiated PC12 cells against Aβ_25–35_-induced apoptosis and tau protein hyperphosphorylation (Cheng et al., 2018). Moreover, the berries of the Chinese plant Schisandra chinensis yield Schizandrol A (SchA), a naturally occurring active ingredient. Prior research has shown that SchA acts as an antioxidant and regulates DNA methylation to ameliorate Aβ_1–40_-induced cell survival and cognitive impairment (Zhang et al., 2015; Hu et al., 2012). According to Song et al., autophagy suppression mediated by the PI3K/AKT/mTOR pathway is responsible for attenuating over-activated autophagy in Aβ_1-42_-induced neurons by SchA, in their experiment on human neuroblastoma SH-SY5Y cells (Song et al., 2020). Nootkatone (Nkt) is extracted from sesquiterpenes in Alpinia oxyphylla, and recent studies indicate that Sch and NKT may work in concert. According to Qi et al., treating Aβ_1–42_-induced PC12 cells with Nkt and Sch stimulated the PI3K/Akt/GSK3β/mTOR pathway, resulting in a neuroprotective effect (Qi et al., 2020). It has been shown that GJ-4 (crocin richments), which is isolated from *Gardenia jasminoides* J. Ellis, targets neuroinflammation and oxidative stress, boosting the cognition of mice treated with Aβ_25-35_ (Zhang et al., 2020). Multiple targets of GJ-4 have been shown in recent investigations, including the Aβ level, tau hyperphosphorylation, and neuroinflammatory reactions. In studies using APP/PS1 transgenic mice, GJ-4 was demonstrated to ameliorate cognitive impairments by preventing neuroinflammation through the PI3K/Akt pathway (Zang et al., 2021). Furthermore, sulforaphane (SF), among the main isothiocyanates found in Raphani Semen, has been demonstrated to possess neuroprotective qualities in AD mouse models. Zhu et al., in their study on adult male SD rats and BV-2 cells, pointed out that SF improved cognitive impairments induced by streptozotocin (STZ) in rats and decreased the AD pathology via modifying the PI3K/Akt/GSK3β pathway (Yang et al., 2020).

The utilization of multi-target pharmaceutical methods is essential in the pharmacotherapy of AD. A considerable literature has grown and supported that traditional Chinese medicines (TCM) contain various components that can prevent or relieve moderate to mild AD through many mechanisms. TCM compound formulations have a special benefit in treating AD as they have multi-target capabilities. Among them, many of the herbal decoctions and formulas act by triggering the PI3K-Akt pathway. Theoretically, there are multiple roles of the PI3K/Akt signaling pathway, so modifications in PI3K activity may result in side effects, which is a negative feature for therapeutical treatments. However, considering the complex components of TCM, side effects are not significant in real experiments. For example, 10 Chinese herbs make up Shenzhiling oral liquid (SZL), a combination used in TCM. Recent investigations state that serum containing SZL has approximately 126 active chemicals, ranging from phenols to phenolic acids. Moreover, SZL treatment was shown by Zheng et al. to exert neuroprotective functions in APP/PS1 mice by reducing A*β*_42_ and Aβ_40_ accumulation in the hippocampi. During this process, the expression of the PI3K/Akt–mTOR signaling pathway has been upregulated (Zheng et al., 2021). Moreover, it has been shown that the 152 active ingredients in Chaihu Shugan San (CSS) can help ameliorate the cognitive impairment linked to AD. According to earlier research, CSS can suppress the production of Bax and prevent inhibitory effect of Aβ on Akt phosphorylation (Zeng et al., 2019).

Natural herbal inhibitors offer a diverse range of options due to their origins in various plants, which may provide a broader spectrum of selectivity for treatment. They are known for their multi-target mechanisms of action, potentially overcoming drug resistance in cancer cells that can develop against single-target inhibitors. These natural inhibitors may also present lower toxicity and fewer side effects, making them safer alternatives to synthetic compounds in some cases.

The cost-effectiveness of natural herbal inhibitors could make them more accessible to patients who might not be able to afford commercially available drugs. Additionally, certain natural compounds have demonstrated immunomodulatory effects that could enhance the immune response of the body to cancer, offering new therapeutic strategies.

However, despite their potential benefits, natural herbal inhibitors face challenges in clinical application, such as standardization, quality control, stability, and long-term safety. While some natural components such as curcumin and resveratrol have shown promise in modulating immune checkpoints in laboratory and animal models, further clinical studies are needed to confirm their efficacy and safety in treating diseases related to GSK3β.

As a GLP-1 agonist, exendin-4 was reported to exhibit neuroprotective activity. Exendin-4 has been shown to reduce levels of Aβ and cholinergic dysfunction in memory-sensitive brain areas of AD rat models, and the action was mediated by the PI3K/Akt pathway (Garabadu and Verma, 2019). In particular, resveratrol (Res) also has neuroprotective properties, and in recent research, it was indicated that a small Resselenium–peptide nanocomposite improves cognitive disorder through various mechanisms, one of which is via preventing Akt from being phosphorylated in brains of AD model mice (Li C. et al., 2021). Evidence abounds that the concentration of some cations in the brain is decreased in AD patients, such as magnesium. As a result, magnesium-L-threonate (MgT), a new magnesium compound, has been applied recently for its capacity to increase brain magnesium levels and reduce degenerative alterations associated with AD. Xiong et al. demonstrated that, in Aβ_25-35_-treated HT22 cells and APP/PS1 mouse hippocampus, MgT treatment exerts neuroprotective benefits against oxidative stress and hippocampal neuronal injury and activates the PI3K/Akt pathway as well (Xiong et al., 2022).

As stated earlier, GSK3β inhibitors are utilized in the treatment of AD because GSK3β is crucial to the pathogenesis of the disease. Lithium has a well-established ability to reduce AD pathogenesis by preventing tau phosphorylation due to its GSK3β inhibitory effects (Haussmann et al., 2021). In terms of clinical efficacy, several recent meta-analyses showed that lithium can successfully enhance cognitive functions in AD patients. According to Shinji et al., there were no appreciable variations in CSF biomarkers between the GSK3β inhibitor and placebo therapy groups. That can be the result of the individual heterogeneity and an inadequate sample size of the group (Matsunaga et al., 2015; Matsunaga et al., 2019). In addition to the many investigations conducted on lithium, we notice that in the past year, several new GSK3*β* inhibitors have been found, synthesized, and produced. In experiments on the HEK293-Tau P301L cell-based model and okadaic acid-induced mouse model, Liu et al. (2023) demonstrated that ZDWX-25, as a harmine derivative, can inhibit both GSK3β and DYRK1A, reducing phosphorylation of multiple Tau epitopes. Synthetic and semi-synthetic GSK3β inhibitors, including 17 β-carboline-1,2,3-triazole hybrids (compound 21), oxazole-4-carboxamide/butylated hydroxytoluene hybrids (compound KWLZ-9e), 1,2,4-thiadiazolidine-3,5-dione derivatives (compound 10a), novel coumarin derivatives (compound 30), and novel *α*-carboline derivatives (compound ZCH-9), exhibit remarkable anti-AD and therapeutic benefits (Liu et al., 2022a; Luo et al., 2023; Dong et al., 2023; Liu et al., 2022b; Chen H. et al., 2022). The drug development strategies focusing on designing inhibitors for single targets based on molecular docking and other technologies represent a future trend in AD treatment.

## 4 Conclusion and future directions

It is well established that PI3K and Akt possess crucial effects on the physiological functions of cells in both the central nervous system and other somatic cells, from neurons and microglia to the myocardium. With insulin, growth factors, and cytokines as upstream modulators and mTOR, GSK3, and FoxO as downstream molecules, PI3K and Akt consist of the whole signaling pathway, maintaining cell homeostasis, and regulating survival, metabolism, apoptosis, inflammation, autophagy, and cytoskeleton remodeling. Abound experimental data within the past decades supported the linkage between the PI3K signaling pathway and AD, the former modulating the pathology of AD including A*β* deposition and tau protein tangles majorly through affecting various processes including inflammation, oxidative stress, cholinergic neurotransmission, synaptic plasticity, and autophagy. Significantly, AD is associated with aberrantly elevated levels of GSK3β, whose inhibitors seem to have intriguing promise as AD treatments. Natural and synthetic modulators are listed in Table 1.

While the genetic determinants of the PI3K pathway are complex and not fully elucidated, the potential influence of expression quantitative trait loci (eQTL) or allele-specific QTL (aQTL) on the variability of gene expression within this pathway is gaining attention. In particular, a recent genome-wide association study (GWAS) has identified 4,166 single nucleotide variants associated with the expression of 85 genes involved in the PI3K/Akt pathway, highlighting the significant role of eQTLs in modulating gene expression within this critical cellular pathway. The study, which utilized mRNA expression data from lymphoblastoid cell lines of 373 Europeans and their genotypes at nearly 6 million nucleotide variants, revealed 73 eQTLs associated with the expression of multiple genes. These eQTLs include variants for genes encoding collagen type I alpha 1 (COL1A1), integrin alpha 11 (ITGA11), and type IV collagen molecules, suggesting a complex regulatory network influenced by genetic variation. Furthermore, the identification of eQTLs in regulatory regions, such as the promoter of the palladin (PALLD) gene and an enhancer of cadherin related family member 3 (CDHR3) gene, points toward a direct impact of these genetic variants on gene expression. This could potentially affect critical cellular processes, including cell adhesion and cytoskeletal organization (Ryu and Lee, 2017). This evidence underscores the importance of considering eQTLs and aQTLs in future studies aiming to dissect the genetic control of the PI3K pathway and its implications in cancer susceptibility and therapy response. By integrating these findings with functional genomics approaches, we can expect to uncover a deeper understanding of the regulatory mechanisms governing the PI3K pathway and identify novel therapeutic targets.

The present review emphasizes that the targets mentioned are valuable to research not only in animal models but also in preclinical and clinical trials, and combinations of multi-target therapeutics and more precise delivery methods, apart from oral administration, are awaiting further exploration.
